# Validation of the Brazilian Portuguese language version of the facial feminization surgery outcomes evaluation the Brazilian Portuguese language version of the FFSOE

**DOI:** 10.1016/j.bjorl.2024.101483

**Published:** 2024-08-03

**Authors:** Vanessa Silva Soledade, Ludimila Sobreira Sena, Lucas Gomes Patrocínio

**Affiliations:** a Clínica Otoface, Uberlândia Medical Center, Uberlândia, MG, Brazil; b Clínica Otoface, Uberlândia Medical Center, Departamento de Otorrinolaringologia e Plástica Facial, Uberlândia, MGN, Brazil

**Keywords:** Transgender woman, Facial surgery, Gender-affirming surgery

## Abstract

**Objective:**

To translate and validate the Facial Feminization Surgery Outcomes Evaluation in Brazilian Portuguese.

**Methods:**

A standard back-translation method was applied to obtain the final Brazilian Portuguese language version. In total, 21 Brazilian native Portuguese speakers transgender women with surgical intent and 21 Brazilian native Portuguese speakers transgender women without surgical intent were included in the study. The FFSOE was administered to both groups and all patients underwent a test-retest two weeks later.

**Results:**

The FFSOE showed a high internal consistency with Cronbach’s alpha greater than 0.8. The test-retest reliability was high with repeated measures being highly correlated in all items, except for item 3, where the difference was significant (*p* =  0.027).

**Conclusion:**

The FFSOE showed reliability, internal consistency and reproducibility in the evaluations. It is easy to understand and quick to apply, making it a simple tool for pre- and post-operative assessment in facial feminization surgeries.

**The Oxford 2011 Levels of Evidence:**

Level 4.

## Highlights

• Practical quality of life assessment tool for transgender women in Brazilian Portuguese.
• Assessment of quality of life through PROMs in transgender women.
• Post-operative assessment in facial feminization surg.

## Introduction

Human sexuality is influenced by biological, psychological and social factors, and is divided into identity, expression, sexual orientation and biological sex. Gender identity is the individual’s perception of themselves. Gender expression is the social construction of the image. Sexual orientation is how individuals relate to each other, which includes sexual attraction. Finally, biological sex is the set of chromosomal information that determines genitalia at birth.

Gender incongruence refers to people whose gender identification differs from those registered at birth. The term transgender is preferable to transsexual because it covers both people who have undergone surgical treatment and those who have not. This is because transgender people can be confident about their identity, without necessarily requiring a medical intervention for physical adjustment to affirm their gender.^1^

On the other hand, some transgender women seek facial feminization surgeries to adapt their features to their identifying gender.^2^, ^3^ The search is more prevalent among those who experience discomfort and anguish related to the divergence between their gender identity and biological sex ‒ a concept defined as gender dysphoria.^4^

The FFS are important in the field of transsexual health because of their impact on self-acceptance and because of their role in social inclusion.^5^ The main objective is to increase the patient's social confidence and reduce gender dysphoria, which is why it is important to use questionnaires to assess satisfaction with the surgical results.^2^, ^6^, ^7^, ^8^, ^9^ In this context, PROMs (Patient-Reported Outcome Measures) are applied ‒ an assessment centered on the patient's condition experience and correlated with higher individual contentment.^10^, ^11^, ^12^

Facial Feminization Surgeries (FFS) are a group of procedures that address facial features, softening them and making them more feminine and harmonious, which is only possible through anatomical and anthropomorphic knowledge that defines “feminine” and “masculine”. The main areas that contribute to facial perception are the nose, frontal region, eyebrows, chin, and jaw. Combined procedures or even more than one approach are often necessary to achieve a satisfactory result for the patient, as highlighted by Morrison et al.^13^

With the growing number of procedures and the lack of instruments for evaluating these patients,^5^, ^14^ there is a need for studies that present an applicable and reliable instrument for this purpose. Given this, this study aimed to translate and validate the Facial Feminization Surgery Outcomes Evaluation (FFSOE) questionnaire into Brazilian Portuguese.^13^, ^14^, ^15^

## Methods

The present study was approved by the Institutional Ethics Committee (6.315.832) and a free and informed consent form was obtained and signed by all participants in the study. The translation process and cultural adaptation were conducted according to the International Society for Pharmacoeconomics and Outcomes Research (ISPOR) guidelines.^12^ This prospective validation study was conducted in two phases. The questionnaire was first translated, and cross-cultural adaptation was carried out. The second phase consisted of the psychometric validation of the questionnaire.

### Questionnaire description

The FFSOE is a questionnaire of 9 statements about the personal perception of facial appearance, including the personal perception of the evaluation of third parties (family members and people close to them) and the interference of appearance with social and professional activities. The instrument uses a five-point Likert scale for the answers (with 0 corresponding to “not at all” and 4 corresponding to “completely”).

### Translation process

The original FFSOE questionnaire (Table 1) was translated into Brazilian Portuguese by 2 independent translators, whose native language is Brazilian Portuguese and who are fluent in English. They were then analyzed by the researchers and reconciled into a third version (final version), considering the context and cultural adaptation. Finally, a third independent translator, a native American and fluent in Portuguese, re-translated the questionnaire into English and compared it to the original version to rule out any loss of concepts or discrepancies with the original questionnaire.

**Table 1:** Facial Feminization Surgery Outcomes Evaluation (FFSOE) – English Version.

Facial Feminization Surgery Outcomes Evaluation (FFSOE)				
1. I like the appearance of my face.				
Not at all	Somewhat	Moderately	Very Much	Completely
2. The appearance of my face is feminine.				
Not at all	Somewhat	Moderately	Very Much	Completely
3. My friends and loved ones perceive my face as feminine.				
Not at all	Somewhat	Moderately	Very Much	Completely
4. My current facial appearance limits my social activities.				
Never	Rarely	Sometimes	Usually	Always
5. My current facial appearance limits my professional activities.				
Never	Rarely	Sometimes	Usually	Always
6. In public I am confident my facial appearance is perceived as feminine.				
Not at all	Somewhat	Moderately	Very Much	Completely
7. I would like to alter the appearance of my face.				
Not at all	Somewhat	Moderately	Very Much	Completely
8. Facial feminization surgery is/was important to my ability to live as a woman.				
Not at all	Somewhat	Moderately	Very Much	Completely
9. Body surgery is/was important to my ability to live as a woman.				
Not at all	Somewhat	Moderately	Very Much	Completely

Interviews were then conducted with ten transgender women who were native speakers of Brazilian Portuguese. During the 15-minute interviews, the first author, a native speaker of Portuguese and fluent in English, reviewed each questionnaire with the patients to identify ambiguities and check its comprehensibility and acceptability. The patients were then asked to verbalize their perception of each item in the three questionnaires. Written notes were taken during the interviews to compile the answers. The data collected was reviewed and modifications were made to the translated versions. A final review was then carried out and a final version was drawn up.

### Application process

The subjects were assessed at a reference outpatient clinic for transgender patients and an outpatient clinic specialized in FFS. Transgender women, over 18 years of age and with Brazilian Portuguese as their native language were included in the study. Transgender women under the age of 18 or who did not have Portuguese as their native language were excluded from the study. In total, 21 native Brazilian transgender women that had facial feminization surgery scheduled and 21 native Brazilian transgender women whom facial feminization surgery was not a personal desire were evaluated. The FFSOE questionnaire was administered to both groups and all participants were retested two weeks later.

### Data analysis

Initially, the normality of the data was tested using the Kolmogorov-Smirnov test. To assess the difference between the first and second responses, two statistical tests were carried out: the Wilcoxon test (intra-group assessment) and the Mann–Whitney test (inter-group assessment). Spearman's correlation, together with the 95% Confidence Interval (95% CI), was carried out to assess the correlations between the volunteers' first and second responses. To measure the reliability and internal consistency of the data collection instrument, Cronbach's alpha was calculated.

Statistical analyses were carried out using SPSS software version 20.0 (SPSS Inc., Chicago, IL, USA), and *p* < 0.05 was considered statistically significant.

## Results

The first stage of the study resulted in the translation and cultural adaptation of the FFSOE questionnaire. The final version in Brazilian Portuguese is shown below (Table 2).

**Table 2:** Facial Feminization Surgery Outcomes Evaluation (FFSOE) – Brazilian Portuguese Version.

Avaliação dos resultados da cirurgia de feminização facial (FFSOE)				
1. Eu gosto da aparência do meu rosto.				
Nem um pouco	Ligeiramente	Moderadamente	Muito	Completamente
2. A aparência do meu rosto é feminina.				
Nem um pouco	Ligeiramente	Moderadamente	Muito	Completamente
3. As pessoas próximas a mim percebem meu rosto como feminino.				
Nem um pouco	Ligeiramente	Moderadamente	Muito	Completamente
4. Minha aparência facial atual limita minhas atividades sociais.				
Nem um pouco	Ligeiramente	Moderadamente	Muito	Completamente
5. Minha aparência facial atual limita minhas atividades profissionais.				
Nem um pouco	Ligeiramente	Moderadamente	Muito	Completamente
6. Em público, eu tenho certeza de que minha aparência facial é percebida como feminina.				
Nem um pouco	Ligeiramente	Moderadamente	Muito	Completamente
7. Eu gostaria de alterar a aparência do meu rosto.				
Nem um pouco	Ligeiramente	Moderadamente	Muito	Completamente
8. A cirurgia de feminização facial é ou foi importante para a minha capacidade de viver como mulher.				
Nem um pouco	Ligeiramente	Moderadamente	Muito	Completamente
9. A cirurgia corporal é ou foi importante para a minha capacidade de viver como mulher.				
Nem um pouco	Ligeiramente	Moderadamente	Muito	Completamente

Table 3, Table 4 show the comparative results of the first and second assessments. No statistically significant differences were found in the responses between the D0 and D14 moments when the individuals were assessed according to groups or together (Table 3). When the groups were compared, there was a difference in the volunteers' answers to question number 3 (Table 4).

**Table 3:** Comparison of intra-group answers, Wilcoxon test.

Question	Group with scheduled surgery (n = 21)	Group without surgical desire (n = 21)	All (n = 42)
	*p*-value	*p*-value	*p*-value
1	0.655	1.000	0.705
2	0.564	0.739	0.564
3	0.083	0.317	0.705
4	0.564	0.157	0.132
5	0.157	0.157	0.527
6	1.000	0.257	0.257
7	0.083	0.527	0.782
8	0.317	1.000	0.564
9	0.317	0.317	0.655

**Table 4:** Comparison of answers between groups, Mann–Whithney test.

Question	Group with scheduled surgery X Group without surgical desire (n = 42)
	*p*-value
1	0.616
2	0.707
3	0.027
4	0.738
5	0.220
6	0.353
7	0.157
8	0.081
9	0.155

Spearman’s correlation analysis between the first and second evaluations found strong/very strong corrections in most of the questions in the questionnaire (Table 5).

**Table 5:** Correlation between the first and second answer, Spearman’s correlation.

Question	Group with scheduled surgery (n = 21)		Group without surgical desire (n = 21)		All (n = 42)	
	*r*	*p*	*r*	*p*	*r*	*p*
1	0.922	<0.001	0.983	<0.001	0.960	<0.001
2	0.944	<0.001	0.856	<0.001	0.904	<0.001
3	0.960	<0.001	0.965	<0.001	0.957	<0.001
4	0.960	<0.001	0.777	<0.001	0.878	<0.001
5	0.978	<0.001	0.779	<0.001	0.876	<0.001
6	1.000	‒	0.906	<0.001	0.958	<0.001
7	0.813	<0.001	0.575	0.006	0.653	<0.001
8	0.999	<0.001	0.939	<0.001	0.955	<0.001
9	0.999	<0.001	0.896	<0.001	0.934	<0.001

Finally, a high level of reliability and internal consistency of the evaluation instrument was observed, with Cronbach's alpha values higher than 0.8 being found for all questions (Table 6).

**Table 6:** Evaluation of the reliability and internal consistency of the instrument, Cronbach's alpha test.

Question	Group with scheduled surgery (n = 21)	Group without surgical desire (n = 21)	All (n = 42)
	*p*-value	*p*-value	*p*-value
1	0.965	0.989	0.979
2	0.974	0.921	0.948
3	0.961	0.974	0.971
4	0.986	0.884	0.942
5	0.991	0.896	0.945
6	1.000	0.953	0.978
7	0.947	0.873	0.893
8	0.983	0.987	0.987
9	0.977	0.966	0.969

## Discussion

The growing number of studies on transsexual health has prompted us to carry out a comprehensive assessment of the quality of life in this population.^5^ The gold standard for this assessment is through the self-assessment quality of life questionnaires, the PROMs, because they provide us with the patient's self-perception of their health condition.^1^, ^6^, ^9^, ^10^

The FFS have become more common, with an increase of 162% between 2015 and 2020, and are an important part of gender affirmation and the treatment of body dysphoria.^14^ In this context, it is essential to have a tool to assess the impact of surgery on the quality of life of transgender women. Satisfaction with the surgical result and the interference of appearance in social and professional activities are equally relevant topics in the evaluation. The instrument also serves as an evaluation standard for comparability in future studies.^10^, ^11^

We believe that this study was successful in translating and adapting the questionnaire into Portuguese, with this version being conceptually equivalent to the original English version. This was only possible due to the multiple stages of translation based on an international protocol. This is important not only because of the semantic translation but also because of the conceptual and cultural preservation.^15^

The Portuguese version is reliable for application, as demonstrated by the high level of reliability and internal consistency found in Cronbach's alpha coefficient. In the re-test phase, we ensured the reproducibility of the instrument, demonstrated by the strong or very strong correlation in Pearson's correlation analysis between the first and second evaluations.

There was a significant difference between the groups on item 3, which asks about the perception of third parties about the patient's appearance. We believe that this difference is justified both by the individual experience of perceiving “feminine” and “masculine”, as well as by the social influence of traditional gender definitions, but also by the experiences that the individual is exposed to daily, including discrimination and judgment of their appearance, which generates feelings of discomfort and makes them perceive one or another characteristic in themselves more. Another consideration regarding the difference in response between the groups is related to the extent to which the opinion of third parties influences the surgical decision.

A limitation related to the study is the relatively small sample size, but with the high correlation found, we believe that a larger sample would reinforce our results. Samples with similar numbers of participants have been used by other authors, such as SCHNOS and Utrecht, with satisfactory statistical quality.

This is the first study to translate and apply a questionnaire in Brazilian Portuguese that assesses this specific population. As already mentioned, the PROMs are of undeniable relevance in assessing quality of life, as well as standardizing the assessment, making the results internationally comparable.

## Conclusion

The FFSOE showed reliability, internal consistency, and reproducibility in the evaluations. It is easy to understand and quick to apply, making it a simple tool for pre- and post-operative assessment in facial feminization surgeries.

## Funding

This research did not receive any specific grant from funding agencies in the public, commercial, or not-for-profit sectors.

## Conflicts of interest

The authors declare no conflicts of interest.
